# The importance of effective data sharing and reuse to funders and others supporting research

**DOI:** 10.1002/leap.1443

**Published:** 2022-01-19

**Authors:** Aki MacFarlane

**Affiliations:** Wellcome Trust

The author is employed by the Wellcome Trust. For the purpose of open access, the author has applied a CC BY public copyright licence to any Author Accepted Manuscript version arising from this submission.

This is the accepted version of the following article: MacFarlane, A. (2022), The importance of effective data sharing and reuse to funders and others supporting research. Learned Publishing, 35: 71-74. which has been published in final form at https://doi.org/10.1002/leap.1443

Research data are often one of the key outputs of a research project, however as many as 30% of articles published in the last decade have no data shared alongside them (Tedersoo *et al*., 2021). There are many discipline-specific repositories available such as Protein Data Bank and the Qualitative Data Repository, as well as general purpose repositories like Zenodo, Figshare and Dataverse. The value to funders of sharing data is relates to a desire to get the greatest possible amount of value from the funding given out. However, as evidenced by The State of Open Data 2020 (Digital Science, 2020) a majority of researchers still have problems or concerns with data sharing.

Funders are increasingly moving towards explicit policy requirements that, wherever possible, data underpinning research articles be available to other researchers at the point of publication. While published articles are important narrative pieces explaining the research project from inception to results, the ability to access the data underpinning the article’s conclusions is incredibly valuable too. It not only enables others to assess the work more fully, but it also provides the opportunity for other researchers to build on this existing data by re-using it.

Secondary data re-use offers great potential for reducing research waste - ensuring that researchers aren’t spending unnecessary time and resources collecting and curating data, which already exists. For example, Savage & Vickers (2009) identified 10 research articles, the use of whose data would have allowed the authors to test a specific pre-specified hypothesis, and requested the data from the authors. They received only 1 dataset in response. Organisations who fund research, whether they be philanthropic or using public funds, have a duty to ensure their funds are being used in the most efficient ways possible and that maximum value is generated from the research they support. Enabling the effective re-use of data holds the potential to speed the progress of research and amplify the resulting benefit. These are compelling arguments for why championing data re-use should be a key priority for all research funders.

At Wellcome, we have a policy that is designed to support the researchers we fund in maximising the value of their research outputs, including data, software and materials (Wellcome, 2017a). We require grant applicants to complete an Outputs Management Plan at the point of application, and encourage them to maintain this as a living document throughout the lifetime of their grant (Wellcome, 2017b). Over recent years - as requirements for such plans have become more commonplace and research institutions have provided increasing support for data management (Teperek & Dunning, 2018, Angelaki & Jones, 2019) – it has been pleasing to see the quality of these plans steadily improve. In September 2020, Wellcome initiated a pilot to provide a support service to further support our funded researchers and institutions to improve the quality of Output Management Plans. Wellcome also works to develop and sustain the infrastructures required to support the quality and longevity of data sharing. We have provided long-term funding to several key data repositories, databases and tools, for example the Single Cell Gene Expression Atlas (Papatheodorou et al., 2020), and are working actively with other funders through fora such as the Global Biodata Coalition to try to ensure such resources have long-term sustainable funding (Anderson, 2017).

However, these activities all focus on encouraging and supporting the sharing of data, and not on stimulating its re-use. Our policy refers to our expectation that research data be re-used in a responsible manner, but we felt our activities lacked an explicit focus on highlighting and encouraging data re-use. We looked to some other examples of incentives for data re-use for inspiration, for example the Economic and Social Research Council’s Secondary Data Analysis Initiative, which is an open call for grant proposals (UK Research and Innovation, 2021), and the New England Journal of Medicine’s SPRINT Data Analysis Challenge, which offered a cash prize for novel findings based on the dataset underlying the SPRINT clinical trial, as well as the opportunity to publish in NEJM (NEJM, 2017).

We launched the Wellcome Data Re-use Prizes in antimicrobial resistance and malaria in November 2018 to reward either new insights or tools that help other researchers to re-use data (Wellcome, 2018). We ran the two prizes concurrently, with each focusing on an area of strategic importance to Wellcome at the time: antimicrobial resistance, and malaria. Entrants were asked to generate a new insight, tool or health application from the available data, and the winner of each prize received £15,000 with 2 runners-up each receiving £5,000. We also offered the winners the opportunity to publish in Wellcome Open Research.

## The antimicrobial resistance prize

This prize highlighted the AMR Register, an open data resource launched by Wellcome’s Drugresistant Infections programme and led by the Open Data Institute. The register has collected information from AMR surveillance programmes generated by the pharmaceutical industry. The antimicrobial resistance prize specifications and all entries are available through Synapse (Synapse, 2019a).

## The malaria prize

This prize highlighted the Malaria Atlas Project, a Repository of Open Access Data (ROAD-MAP), launched with support from Wellcome, and then funding from the Bill & Melinda Gates Foundation. The repository contains a wealth of data on malaria risk and intervention coverage. The malaria prize specifications and all entries are available through Synapse (Synapse 2019b).

The judging panels were impressed by the calibre of the entries we received to both prizes, but there were lessons to be learned from this endeavour. We were keen to market this prize to PhD students or postdocs, targeting researchers early in their careers in the hope of embedding data re-use skills and enthusiasm in their research, and were pleased that two of the prize winners were individual PhD students, and one a whole team of PhD students, with PhD students involved in other winning entries too. However, we did not receive the volume of applications from our target audience as we’d hoped, and received feedback that the generosity of the prizes we offered was disproportionately large compared to the type of findings or tools we were hoping to see, and therefore many prospective entrants felt they wouldn’t have the time to produce a worthy entry. And at the end of the day, positioning these prizes as work that could be done alongside researchers’ or students’ existing workloads may have inadvertently sent a message that data re-use is “nice to have” rather than a crucial part of an effective research enterprise.

Of course, in order to be viable, initiatives such as these rely on relevant data being available, having untapped potential, and being easily reusable i.e. well curated and annotated. But for data to be reusable, it must first be findable and accessible! Published articles are a useful signpost to the existence of data. Publishers have a key role to play here by prompting researchers to ensure that the data they share are discoverable and reusable. The State of Open Data 2020 (Digital Science, 2020) reported that when researchers were asked which source they would rely on for help making data from their most recent research report open, “Publisher” was the most commonly selected response. Some examples of actions that publishers could take are:

1. Requiring data availability statements,
2. Requiring peer reviewers to comment on the data availability statements,
3. Querying data availability statements that indicate data can only be obtained by contacting the researcher directly, and push for data to be available online (with access management if appropriate),
4. Encouraging the use of recognised community repositories where these exist, and maintain a list of recommended repositories,
5. Requiring authors to include persistent identifiers for their data in the data availability statement where they have been able to obtain one.

And in turn, as a global health research funder, Wellcome is committed to continuing to trial innovative funding approaches, and to working with the research community and other stakeholders to unlock the full potential of data to improve global health – recognising that many issues are common across all fields of research and that there is much to be learned from other disciplines.

## Figures and Tables

**Table 1:** Summary of the winning entries to the Wellcome Data Re-use Prize in Antimicrobial Resistance

	Description	Entrant(s)	Link to submission	Subsequent publication
1^st^ prize	Development of a composite index of antibiotic resistance for common infection syndromes.	Quentin Leclerc, Gwen Knight, Nichola Naylor, Francesc Coll and Alexander Aiken	https://www.synapse.org/#!Synapse:syn18201040/wiki/588540	Wellcome Open Research (Leclerc *et al.,* 2020)
Runne r-up	Reanalysis of the dataset to examine cross-correlation of antibiotic minimum inhibitory concentrations, as well as the correlation of resistance rates with antibiotic consumption.	Liam Shaw	https://www.synapse.org/#!Synapse:syn18344812/wiki/588612	Wellcome Open Research (Shaw, 2020)
Runne r-up	A novel decision-making tool applied in urosepsis, to optimise antibiotic selection.	Zafer Tandogdu, Truls Erik Bjerklund Johansen, Florian Wagenlehner, Kurt Naber and Evgenios Kakariadis	https://www.synapse.org/#!Synapse:syn18377564/wiki/588796	

**Table 2:** Summary of the winning entries to the Wellcome Data Re-use Prize in Malaria

	Description	Entrant(s)	Link to submission	Subsequent publication
1^st^ prize	Studying the causal effect of malaria prevalence on anemia prevalence from the community level using causal inference techniques	Shuxiao Chen, Emily Diana, Sheng Gao, Siyu Heng, Hongming Pu, Hua Wang and Dylan Small	https://www.synapse.org/#!Synapse:syn18379247/wiki/588810	
Runne r-up	An interactive R shiny application for summary statistics and visualisation of Malaria Atlas Project data.	Joshua Longbottom, Andy South and Sean Tomlinson	https://www.synapse.org/#!Synapse:syn18429096/wiki/590185	Wellcome Open Research (Tomlinson *et al.,* 2019)
Runne r-up	Exploring gene drives as an intervention for malaria control in sub-Saharan Africa using modelling approaches based on data from the Malaria Atlas Project	Nawaphan Metchanun	https://www.synapse.org/#!Synapse:syn18409176/wiki/589692	medRxiv (Metchanun *et al.*, 2020)

## Summary bullets

- Funders including Wellcome are working more and more on encouraging data sharing
- Further focus needs to be put on stimulating the re-use of research data once shared
- Wellcome ran data re-use prizes in 2018/19 to incentivise data re-use
- Publishers as well as funders have a key role to play in supporting the research community to share data and maximise its value
